# Use of drug-eluting balloon coronary intervention prior to living donor kidney transplantation

**DOI:** 10.1186/1471-2261-14-112

**Published:** 2014-09-01

**Authors:** Tobias Kammerer, Andres Beiras-Fernandez, Markus Rehm, Manfred Stangl, Markus Guba, Christian Kupatt-Jeremias, Florian Weis

**Affiliations:** Department of Anesthesiology, University of Munich, Großhadern, Marchioninistrasse 15, 81377 Munich, Germany; Department of Thoracic and Cardiovascular Surgery, University Hospital, JW Goethe-University, Theodor–Stern–Kai 7, 61590 Frankfurt, Germany; Department of Surgery, University of Munich, Großhadern, Marchioninistrasse 15, 81377 Munich, Germany; Department of Cardiology, University of Munich, Großhadern, Marchioninistrasse 15, 81377 Munich, Germany; Department of Anaesthesiology – Clinic Fürstenfeldbruck, Dachauer Strasse 33, Fürstenfeldbruck, D-82256 Germany

## Abstract

**Background:**

Kidney transplantation is the gold standard of therapy in patients with terminal renal insufficiency. Living donor transplantation is a well-established option in this field. Enlarging the donor’s pool implicates the acceptance of an increased rate of comorbidities. Among them, coronary artery disease is a growing problem. An increasing number of patients, undergoing living donation, receive antiplatelet therapies due to coronary disease.

**Case presentation:**

Here we report about the perioperative treatment with a drug-eluting balloon in a patient with major cardiac risk factors who underwent kidney transplantation.

**Conclusion:**

At the current time no recommendation can be given for the routine use of drug-eluting balloons.

## Background

Kidney transplantation is still the gold standard of therapy in patients with terminal renal insufficiency. Thus, the criteria for candidates for kidney transplantation have been weakened over the last decades to offer this option to a larger group of patients with otherwise worse outcome. Living donor transplantation is becoming more and more a well-established option in this field. However, clinicians will have to deal more and more with patients suffering from a number of relevant co-morbidities. Especially coronary artery disease (CAD) is becoming a growing problem in perioperative medicine as current standard treatment options (i.e. coronary stenting) often go along with long lasting antiaggregation protocols [1, 2]. Various aspects of antiaggregation after percutaneous transluminal coronary angioplasty (PTCA) are partly discussed controversially [3]. Dual platelet inhibition after placement of drug eluting or bare metal stents may lead to considerable bleeding complications during the surgical intervention [4, 5]. By contrast an interruption of antiplatelet therapy results in a significant increase of perioperative cardiac complications [6, 7]. At the same time, more and more drug-eluting stents are used by interventional cardiologists. To minimise this problem a bipartite intervention using a drug-eluting balloon is a possible alternative [8–10]. Until now, no investigations or case reports are published examining the use of drug-eluting balloons in a perioperative setting. Here we report about the perioperative treatment with a drug-eluting balloon in a kidney transplant patient with major cardiac risk factors.

## Case presentation

A 66-year-old man (height 172 cm, weight 92 kg) presented with diabetic nephropathy for planned living donor kidney transplantation. He suffered from CAD, hypertension, hypercholesterolemia, insulin dependent diabetes mellitus II and a chronic obstructive pulmonary disease. He had undergone coronary artery bypass surgery in a different hospital 7 months before (left internal mammary artery (LIMA) to left anterior descending (LAD) and saphenous vein graft to the ramus diagonalis). The ramus circumflexus (RCX) was not accessible to revascularization during bypass operation according to the documents of the different hospital. The LAD was supplied with a drug-eluting stent three years before. The RCX was supplied with a bare-metal stent one year before and, because of an in-stent restenosis, 5 month before with a Genous stent in a different hospital. Additionally another Genous stent was placed distally to the previous stent area.

**Figure 1:**
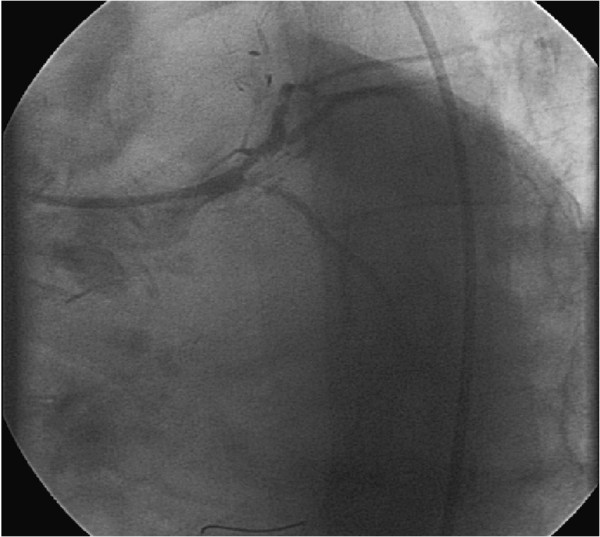
**(preoperative angiography): stenosis of the proximal ramus circumflexus.**

**Figure 2:**
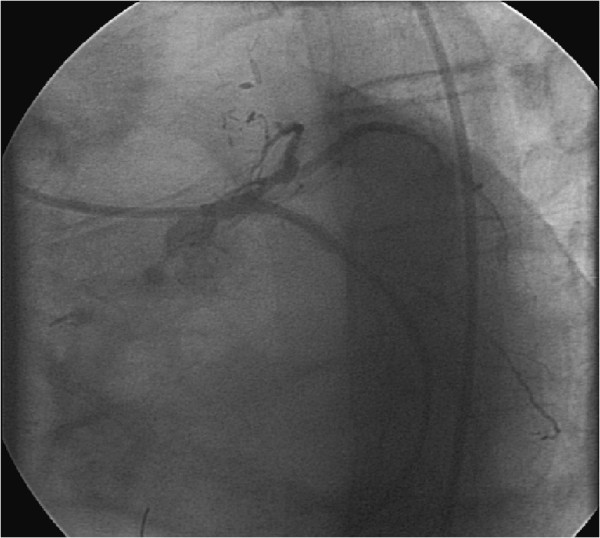
**(preoperative angiography): ramus circumflexus after dilatation with a drug-eluting balloon.**

The patients’ wife was intended to serve as the organ donor. She had a history of severe chronic obstructive pulmonary disease with intercurrent infectious exacerbation, why the transplantation had been called off twice, yet. Prior to the planned date of transplantation she was in good condition.On account of the medical history of the recipient and according to the pre-transplantation guidelines of our institution with regard to high-risk patients a cardiac catheterization was carried out 5 days before transplantation. Here an in-stent restenosis of the proximal ramus circumflexus, which had already been supplied with drug-eluting and bare metal stents two times before, was observed (see Figure 1). After intensive discussion between the cardiologist, the surgeon and the anaesthesiologist the following therapeutic plan was made: the stenosis was dilated with a 3.0/15 mm drug-eluting balloon (Inc. Braun, Germany) followed by anticoagulation with tirofiban and aspirin until surgery. The post-interventional angiography showed a very good result after PTCA (see Figure 2). Definitive stenting was planned after full recovery from the kidney transplantation.

**Figure 3:**
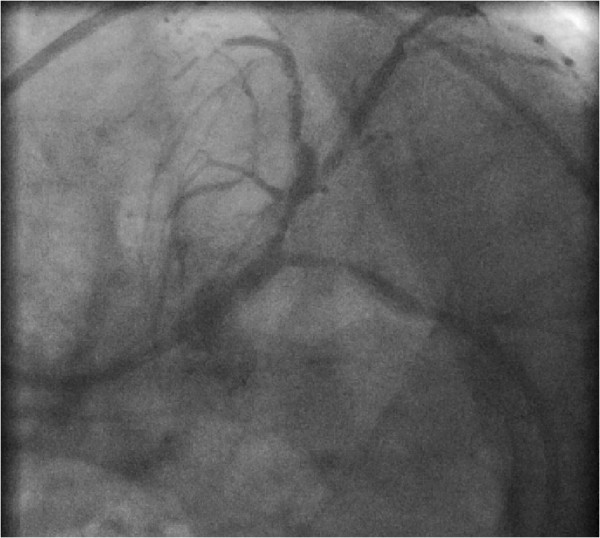
**(postoperative angiography): restenosis of the proximal ramus circumflexus.**

**Figure 4:**
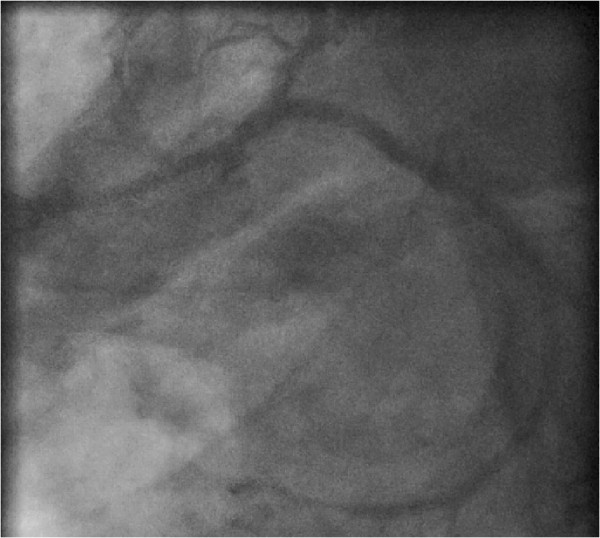
**(postoperative angiography): revascularization of ramus circumflexus after placement of a drug-eluting stent.**

Five days later the planned kidney transplantation was carried out. Medication including aspirin and tirofiban was continued up to the day of operation. In the operating room (OR) induction with sufentanil 0.33 μg kg^-1^, propofol 1.6 mg kg^-1^ and rocuronium 0.33 mg kg^-1^ was performed. Anaesthesia was maintained with sevofluoran 1.6 Vol%. During whole operation mean arterial pressure was above 70 mmHg with a heart rate between 50 and 60 bpm. The patient received norepinephrine with a maximum dose of 0.11 μg kg^-1^ min. Intraoperative blood loss was 800 ml, and the lowest level of haemoglobin was 12.2 g/l. The patient received 2500 ml of crystalloid solution. After successful kidney transplantation he was extubated in the OR and transferred to the intensive care unit (ICU).One hour after admission to the intensive care unit (ICU) the patient developed pectanginous symptoms. The ECG at 10 minutes after clinical presentation of angina revealed no significant abnormalities. The cardiac troponin I was initial negative and 13.9 pg/ml after 4 hours with a peak level of >50.0 pg/ml 7 hours after admission. Cardiac catheterization, carried out 20 minutes after the first complaint of angina by the patient presented a sub-acute in-stent thrombosis of the RCX stent which had been dilated preoperatively with a drug-eluting balloon (see Figure 3). After PTCA with a 2.0 mm balloon the implantation of a 3.0/24 mm drug-eluting stent (Medtronic Endeavor Resolute, Medtronic, Germany) followed. The angiography again showed a good result (see Figure 4). Postinterventionally, after consultation of the surgeons, anticoagulation with tirofiban over 3 days was started followed by prasugrel for 12 months and aspirin. During anticoagulation only minor skin bleeding appeared. 12 days after kidney transplantation the patient could be dismissed home in a good state and is still well after three years.

## Discussion

The perioperative management of patients undergoing non-cardiac surgery in the age of increasing percutaneous coronary intervention is a major problem. The present treatment is based on the recommendations of the American College of Cardiology Foundation (ACCF) and the American Heart Association (AHA) [11, 12]. Additionally, guidelines of the European Society of Cardiology (ESC) and the European Society of Anaesthesiology (ESA) were published in 2009 [13].

Patients, receiving a surgical intervention within the first 35 days after coronary stent implantation, have an increased risk of stent thrombosis with consecutively increased morbidity and mortality [14]. Hence, it is recommended to delay elective interventions about at least 6 weeks (after bare metal stent) [15] or 12 months (after drug eluting stents) [16]. If this is no opinion in urgent cases or emergency surgery, an individual, patient adapted strategy has to be carried out. In the present case, the living donor transplantation was called off twice because of an infect exacerbation of the donor, who was now in a good condition. After interdisciplinary discussion the renewed in-stent restenosis was treated by a drug-eluting balloon, followed by anticoagulation with tirofiban and aspirin until surgery. Eptifibatide [17] or tirofiban [18] is a possible alternative to clopidogrel. If dual antiplatelet therapy is not possible on account of high bleeding risk, for example in neurosurgery, clopidogrel can be stopped 5–7 days before surgery under current medication with aspirin [16]. Instead of stenting with BMS or DES a pure balloon angioplasty should be performed under specific circumstances [16]. These specific circumstances are, however, not specified in the guideline. Thus, preoperative PTCA without stenting seems to be a good “bridging strategy” for patients with intermediate urgency of the planned operation. A recently increasingly used alternative is an intervention with drug eluting balloons [8–10]. Several clinical investigations underline the meaning of drug-eluting balloons in the treatment of instent stenosis [19–21]. This new option may even foster the attitude towards the mentioned “bridging strategy”.

## Conclusions

In our opinion and based on this report the use of a drug-eluting balloon intervention should only be performed under special circumstances: the whole team has to be aware of the potential risk of perioperative myocardial ischemia, and the option of immediate coronary re-intervention by a cardiologist familiar with the respective case has to be well prepared. In the present case it was even the same cardiologist who had performed the initial intervention.

This underlines the meaning of an interdisciplinary approach in the perioperative care of cardiac patients which concerns not only surgeons and anaesthesiologists, but in particular also the treating cardiologists.

On account of the insufficient data at the current time from our point of view no recommendation can be given for the routine use of drug-eluting balloons.

## Patient’s consent

Written informed consent was obtained from the patient for publication of this Case report and any accompanying images. A copy of the written consent is available for review by the Editor of this journal.

## References

[1] Khair T, Garcia B, Banerjee S, Brilakis ES (2011). Contemporary approaches to perioperative management of coronary stents and to preoperative coronary revascularization: a survey of 374 interventional cardiologists. Cardiovasc Revasc Med.

[2] Howard-Alpe GM, de Bono J, Hudsmith L, Orr WP, Foex P, Sear JW (2007). Coronary artery stents and non-cardiac surgery. Br J Anaesth.

[3] Bornemann H, Pruller F, Metzler H (2010). The patient with coronary stents and antiplatelet agents: what to do and how to deal?. Eur J Anaesthesiol.

[4] Kapetanakis EI, Medlam DA, Boyce SW, Haile E, Hill PC, Dullum MK, Bafi AS, Petro KR, Corso PJ (2005). Clopidogrel administration prior to coronary artery bypass grafting surgery: the cardiologist’s panacea or the surgeon’s headache?. Eur Heart J.

[5] Yende S, Wunderink RG (2001). Effect of clopidogrel on bleeding after coronary artery bypass surgery. Crit Care Med.

[6] Collet JP, Montalescot G, Blanchet B, Tanguy ML, Golmard JL, Choussat R, Beygui F, Payot L, Vignolles N, Metzger JP, Thomas D (2004). Impact of prior use or recent withdrawal of oral antiplatelet agents on acute coronary syndromes. Circulation.

[7] Sharma AK, Ajani AE, Hamwi SM, Maniar P, Lakhani SV, Waksman R, Lindsay J (2004). Major noncardiac surgery following coronary stenting: when is it safe to operate?. Catheter Cardiovasc Interv.

[8] Azarisman SM, Sabruddin MZ, Rosli MA (2011). Recurrent in-stent restenosis with total occlusion remedied with drug-eluting balloon angioplasty. Int Heart J.

[9] Habara S, Mitsudo K, Kadota K, Goto T, Fujii S, Yamamoto H, Katoh H, Oka N, Fuku Y, Hosogi S, Hirono A, Maruo T, Tanaka H, Shigemoto Y, Hasegawa D, Tasaka H, Kusunose M, Otsuru S, Okamoto Y, Saito N, Tsujimoto Y, Eguchi H, Miyake K, Yoshino M (2011). Effectiveness of paclitaxel-eluting balloon catheter in patients with sirolimus-eluting stent restenosis. JACC Cardiovasc Interv.

[10] Joost A, Kurowski V, Radke PW (2010). Drug eluting balloons for the treatment of coronary artery disease: what can we expect?. World J Cardiol.

[11] Eagle KA, Berger PB, Calkins H, Chaitman BR, Ewy GA, Fleischmann KE, Fleisher LA, Froehlich JB, Gusberg RJ, Leppo JA, Ryan T, Schlant RC, Winters WL, Gibbons RJ, Antman EM, Alpert JS, Faxon DP, Fuster V, Gregoratos G, Jacobs AK, Hiratzka LF, Russell RO, Smith SC, American College of Cardiology (2002). ACC/AHA guideline update for perioperative cardiovascular evaluation for noncardiac surgery–executive summary: a report of the American College of Cardiology/American Heart Association Task Force on Practice Guidelines (Committee to Update the 1996 Guidelines on Perioperative Cardiovascular Evaluation for Noncardiac Surgery). J Am Coll Cardiol.

[12] Fleisher LA, Beckman JA, Brown KA, Calkins H, Chaikof E, Fleischmann KE, Freeman WK, Froehlich JB, Kasper EK, Kersten JR, Riegel B, Robb JF, Smith SC, Jacobs AK, Adams CD, Anderson JL, Antman EM, Buller CE, Creager MA, Ettinger SM, Faxon DP, Fuster V, Halperin JL, Hiratzka LF, Hunt SA, Lytle BW, Nishimura R, Ornato JP, Page RL, Tarkington LG (2007). ACC/AHA 2007 guidelines on perioperative cardiovascular evaluation and care for noncardiac surgery: a report of the American College of Cardiology/American Heart Association Task Force on Practice Guidelines (Writing Committee to Revise the 2002 Guidelines on Perioperative Cardiovascular Evaluation for Noncardiac Surgery): developed in collaboration with the American Society of Echocardiography, American Society of Nuclear Cardiology, Heart Rhythm Society, Society of Cardiovascular Anesthesiologists, Society for Cardiovascular Angiography and Interventions, Society for Vascular Medicine and Biology, and Society for Vascular Surgery. Circulation.

[13] Poldermans D, Bax JJ, Boersma E, De Hert S, Eeckhout E, Fowkes G, Gorenek B, Hennerici MG, Iung B, Kelm M, Kjeldsen KP, Kristensen SD, Lopez-Sendon J, Pelosi P, Philippe F, Pierard L, Ponikowski P, Schmid JP, Sellevold OF, Sicari R, Van den Berghe G, Vermassen F (2009). Guidelines for pre-operative cardiac risk assessment and perioperative cardiac management in non-cardiac surgery. Eur Heart J.

[14] Vicenzi MN, Meislitzer T, Heitzinger B, Halaj M, Fleisher LA, Metzler H (2006). Coronary artery stenting and non-cardiac surgery–a prospective outcome study. Br J Anaesth.

[15] Kaluza GL, Joseph J, Lee JR, Raizner ME, Raizner AE (2000). Catastrophic outcomes of noncardiac surgery soon after coronary stenting. J Am Coll Cardiol.

[16] Korte W, Cattaneo M, Chassot PG, Eichinger S, von Heymann C, Hofmann N, Rickli H, Spannagl M, Ziegler B, Verheugt F, Huber K (2011). Peri-operative management of antiplatelet therapy in patients with coronary artery disease. Joint position paper by members of the working group on Perioperative Haemostasis of the Society on Thrombosis and Haemostasis Research (GTH), the working group on Perioperative Coagulation of the Austrian Society for Anesthesiology, Resuscitation and Intensive Care (OGARI) and the Working Group Thrombosis of the European Society for Cardiology (ESC). Thromb Haemost.

[17] Wilczynski M, Bochenek T, Goral J, Knast K, Abu Samra R, Wita K, Bochenek A (2009). [Use of eptifibatide in patients with acute stent thrombosis, requiring urgent surgical revascularisation - report of 2 cases]. Kardiol Pol.

[18] Savonitto S, D’Urbano M, Caracciolo M, Barlocco F, Mariani G, Nichelatti M, Klugmann S, De Servi S (2010). Urgent surgery in patients with a recently implanted coronary drug-eluting stent: a phase II study of ‘bridging’ antiplatelet therapy with tirofiban during temporary withdrawal of clopidogrel. Br J Anaesth.

[19] Scheller B, Hehrlein C, Bocksch W, Rutsch W, Haghi D, Dietz U, Böhm M, Speck U (2006). Treatment of coronary in-stent restenosis with a paclitaxel-coated balloon catheter. N Engl J Med.

[20] Scheller B, Hehrlein C, Bocksch W, Rutsch W, Haghi D, Dietz U, Böhm M, Speck U (2008). Two year follow-up after treatment of coronary in-stent restenosis with a paclitaxel-coated balloon catheter. Clin Res Cardiol.

[21] Clever YP, Rosenkranz S, Bohm M, Scheller B (2008). Hotline update of clinical trials and registries presented at the ACC and SCAI-ACCi2 meeting 2008 in Chicago. Clin Res Cardiol.

[CR22] The pre-publication history for this paper can be accessed here:http://www.biomedcentral.com/1471-2261/14/112/prepub

